# Reimbursement Policies of Swiss Health Insurances for the Surgical Treatment of Symptomatic Abdominal Tissue Excess After Massive Weight Loss: A Retrospective Cohort Study

**DOI:** 10.3390/jcm14186617

**Published:** 2025-09-19

**Authors:** Valeria Pruzzo, Francesca Bonomi, Leon Guggenheim, Astrid Navarra, Daniel Schmauss, Reto Wettstein, Yves Harder

**Affiliations:** 1 Department of General Surgery, Ospedale Regionale di Mendrisio, Ente Ospedaliero Cantonale (EOC), 6850 Mendrisio, Switzerland; pruzzovaleria@gmail.com (V.P.); francescabonomi.bonnie@gmail.com (F.B.); 2 Faculty of Biomedical Sciences, Università della Svizzera Italiana, 6962 Lugano, Switzerland; leon.guggenheim@gmx.ch (L.G.); daniel.schmauss@eoc.ch (D.S.); 3 Department of Plastic Surgery and Hand Surgery, Cantonal Hospital Aarau, 5001 Aarau, Switzerland; astrid.navarra@bluewin.ch; 4 Department of Plastic, Reconstructive and Aesthetic Surgery, Ospedale Regionale di Lugano, Ente Ospedaliero Cantonale (EOC), 6900 Lugano, Switzerland; 5 Department of Plastic, Reconstructive, Aesthetic and Hand Surgery, University Hospital of Basel, 4031 Basel, Switzerland; reto.wettstein@hin.ch; 6 Department of Plastic, Reconstructive, Aesthetic Surgery and Hand Surgery, Centre Hospitalier Universitaire Vaudois (CHUV), 1005 Lausanne, Switzerland; 7 Faculty of Biology and Medicine, University of Lausanne (UNIL), 1005 Lausanne, Switzerland

**Keywords:** abdominoplasty, abdominal tissue excess, health insurance company, massive weight loss, reimbursement policy

## Abstract

**Background**: Patients with symptomatic abdominal tissue excess following massive weight loss (MWL) often experience skin affections associated with hygiene challenges, functional impairments, and psychological distress, all of which significantly impact their quality of life (QoL). Abdominoplasty effectively addresses these issues when conservative treatments prove ineffective. However, health insurance companies (HICs) in Switzerland frequently deny reimbursement. This study aimed to evaluate HIC’s reimbursement policies for abdominoplasty, quantifying time delays and additional costs generated by reconsideration due to initial rejections while assessing postoperative QoL of patients. **Methods**: A retrospective cohort study was conducted including patients undergoing abdominoplasty for symptomatic abdominal tissue excess after MWL between July 2019 and December 2023. Eligibility required HIC approval, informed consent, and legal age. Primary outcomes measured the number of reimbursement requests needed per patient, duration until approval, and additional diagnostic and therapeutic interventions following rejection. Secondary outcomes focused on additional consequent costs, differences in baseline characteristics and symptomatology, as well as QoL improvements using a non-validated, study-specific questionnaire. **Results**: Of 52 patients included, 33 received cost approval after a single request, whereas 19 required multiple submissions. The mean duration until approval was 15 weeks, with a 26-week delay for the multiple-request group, generating additional costs of CHF 715 per patient. Moreover, abdominoplasty significantly improved QoL in all patients, with no differences between groups. **Conclusions**: Initial reimbursement denials caused treatment delays, prolonged symptomatology, and increased healthcare costs, despite clear surgical indications. However, future studies involving larger cohorts are needed to support these findings.

## 1. Introduction

Obesity represents one of the most significant global health challenges, with its prevalence steadily increasing worldwide. By 2030, it is estimated that approximately 42% of the global population will be classified as obese [1]. This condition is closely associated with a range of comorbidities, including diabetes, cardiovascular diseases, degenerative joint disorders, and other health complications [2]. In response to this growing epidemic, various weight loss strategies have been developed, such as physical activity, dietary modifications, bariatric surgery, and more recently weight loss injections initially intended as a medical aid for managing type 2 diabetes [3,4,5]. Among these, bariatric surgery so far has emerged as the most successful and durable treatment for achieving massive weight loss (MWL) [6], internationally defined as a loss of 50% or more of excess body weight [7,8], improving health-related quality of life (QoL) [9,10].

However, a major consequence of successful MWL is the development of excess tissue, affecting up to 96% of patients [11]. This redundant tissue is most frequently located on the abdomen, the upper arms, the medial thighs, and the breasts [12,13]. Symptoms and signs associated with the abdominal skin apron are varied and consistently reported, including scars and back pain, functional impairments, as well as hygiene challenges potentially inducing skin irritations, ranging from cutaneous rashes to severe soft tissue infections [14]. If conservative treatments (e.g., moisture-absorbing agents, analgesics, antifungals, antibiotics, physiotherapy and/or acupuncture sessions) remain ineffective in the long term, surgery may become necessary. Abdominoplasty has demonstrated effectiveness in addressing these functional health issues associated with MWL-related excess tissue [14,15,16].

In Switzerland, residents are required to obtain compulsory basic health insurance from an accredited health insurance company (HIC). According to the Swiss Federal Health Insurance Act (KVG/LAMal), HICs are obliged to cover the costs for any procedure deemed necessary to diagnose or treat a disease or its sequelae [17]. Swiss social security law states that all medical procedures and treatments must be effective, appropriate, and economical [18]. Regarding the necessity of abdominoplasty, the HIC’s trusted physician determines patient eligibility for reimbursement based on the board-certified plastic surgeon’s out-patient evaluation that is reproduced in a letter and accompanied by representative photographic documentation, assessing whether there is a demonstrable ‘value of disease’ and eventually an obligation for reimbursement. This evaluation is conducted in accordance with the Swiss Diagnosis Related Group (DRG) criteria for cost assumption (Table 1) [18].

Despite the relatively well-structured legal criteria, they appear to allow for a degree of ‘interpretative flexibility’. This often results in the refusal of cost coverage despite the indication of the board-certified plastic surgeon. Many of these requests are ultimately approved after multiple submissions, resulting in unnecessary delays and wasted resources [19]. Therefore, this study aims to evaluate and critically discuss reimbursement policies for the surgical treatment of symptomatic abdominal tissue excess following MWL, with a particular focus on their impact on treatment delays, including the potential need for additional diagnostic and therapeutic interventions and the consequent increase in healthcare costs.

## 2. Materials and Methods

### 2.1. Study Design and Participants

A retrospective descriptive cohort study was performed, including patients who underwent abdominoplasty for symptomatic abdominal tissue excess secondary to MWL between July 2019 and December 2023. In this study, the term ‘abdominoplasty’ encompassed all surgical procedures treating symptomatic abdominal tissue excess, such as abdominoplasty, belt lipectomy/lower body lift and panniculectomy [20]. Eligibility requirements included cost approval by the HIC according to Swiss DRG criteria and informed consent. The exclusion criteria were as follows: (1) self-paying patients; (2) asymptomatic patients and those without MWL, defined as a loss of ≥50% of excess body weight; (3) patients undergoing abdominal tissue resection for microvascular flap-based breast reconstruction; and (4) patients younger than 18 years.

All patients were evaluated by a board-certified plastic surgeon, who assessed the need for abdominoplasty on a case-by-case basis, to address the underlying pathology. During the study period, the department has employed a total of 6 board-certified plastic surgeons, with the head of the department being the only consistent evaluator. A reimbursement request letter was submitted to the HIC including standard photographs, frequently accompanied by supporting documentation such as reports from other specialists or evaluations of radiological imaging. Once cost approval was granted, the surgery was performed by the same board-certified plastic surgeon who had evaluated the patient initially. Postoperative follow-up evaluations were usually performed at 2, 6, 12 weeks, as well as 6, 12 months, and then yearly (mean follow-up 12 months; range 1–65 months). These evaluations included the assessment of the patient’s medical history and a comprehensive clinical examination.

At the time of the last follow-up visit, all patients were interviewed using a non-validated questionnaire, specifically designated for this study, including the assessment of surgery-induced changes on recurrent skin affections, functional impairments (such as mobility restrictions and hygiene challenges), psychological disorders, pain at the abdominal apron and back pain. The different variables were scored according to a 10-point numerical rating scale (NRS) where higher scores indicated a greater impact on patient’s QoL. The average score of the individual variables was considered an overall assessment of the patient’s QoL before and after surgery.

### 2.2. Primary and Secondary Outcomes Measures

Primary outcomes included the number of written requests and reconsiderations required to achieve cost approval from the HIC. Patients were categorized into two groups: those who received cost approval directly after a single request (single-request group) and those requiring two or more requests (multiple-request group). Furthermore, the mean duration of the approval process (in weeks) from the initial reimbursement request to the receipt of cost approval was evaluated. Finally, the time difference between the two groups was calculated, representing the additional ‘delay’ time (in weeks) in patients with multiple requests. Another primary outcome concerned the number and types of additional out-patient visits required between the first request and cost approval, respectively, between the initial rejection and the final acceptance. These included further evaluations by plastic surgeons, as well as consultations with other specialists. Moreover, diagnostic procedures (e.g., ultrasound and computed tomography (CT) scans) and conservative treatments were also considered in the analysis.

Secondary outcomes included the additional costs (in CHF) incurred by the multiple-request group compared to the single-request group. TARMED is the standardized Swiss individual tariff structure for out-patient medical services used for billing healthcare services provided to external patients. Accordingly, predefined TARMED-based prices were applied to calculate the amount of additional costs (Table 2).

To provide a comprehensive baseline for patient analysis, the following demographic parameters were collected: gender, age at surgery (years), comorbidities, class of health insurance (i.e., class III (compulsory), class II or class I (complementary)), height (cm), body mass index (BMI) (kg/m^2^), weight before MWL (kg), weight at the time of abdominoplasty (kg), ideal weight (kg), excess body weight before MWL (kg) as well as methods to achieve MWL (e.g., gastric bypass, sleeve gastrectomy, gastric banding or diet) and duration of weight stability after MWL (months). Additional evaluations included the presence of skin affections such as rashes or ulcerations, psychological disorders, functional impairments, back pain, symptomatic rectus diastasis, and abdominal hernia associated with MWL. Moreover, the severity of abdominal deformity was retrospectively assessed using preoperative photographs and classified according to the Pittsburgh Rating Scale [21].

For each patient, the specific surgical body contouring procedure performed to address abdominal excess (e.g., abdominoplasty, belt lipectomy/lower body lift, and panniculectomy) was recorded, along with the weight (kg), and surface area (%) of the resected abdominal tissue. This area was calculated by dividing the weight of resected abdominal tissue by the product of an estimated average flap thickness of 2 cm and the assumed flap density (approximated to that of water, i.e., 1000 kg/m^3^). The percentage (%) of the resected tissue surface was obtained by dividing the previously determined flap surface area (cm^2^) by the body surface area (BSA, m^2^) of an average individual (170 cm, 70 kg), which was calculated using the Mosteller formula: BSA = √ [(height (cm) × weight (kg))/3600]. Moreover, eventual self-funded accompanying procedures performed during the same surgical session (e.g., mastopexy, cruroplasty, brachioplasty, and treatment of rectus diastasis) and the length of the postoperative hospital stay were considered.

Finally, the QoL of patients in both groups before and after abdominal excess resection was compared using the abovementioned designated questionnaire.

### 2.3. Statistical Analysis

Statistical analysis was performed using R version 4.4.2. The data was initially tested for normal distribution and equal variance. Differences between the two groups were assessed by *t*-test, Mann–Whitney-U-Test, paired Wilcoxon signed-rank test or Fisher’s exact test as appropriate. All tests were performed two-sided and a *p*-value of <0.05 was considered statistically significant.

## 3. Results

**Figure 1:**
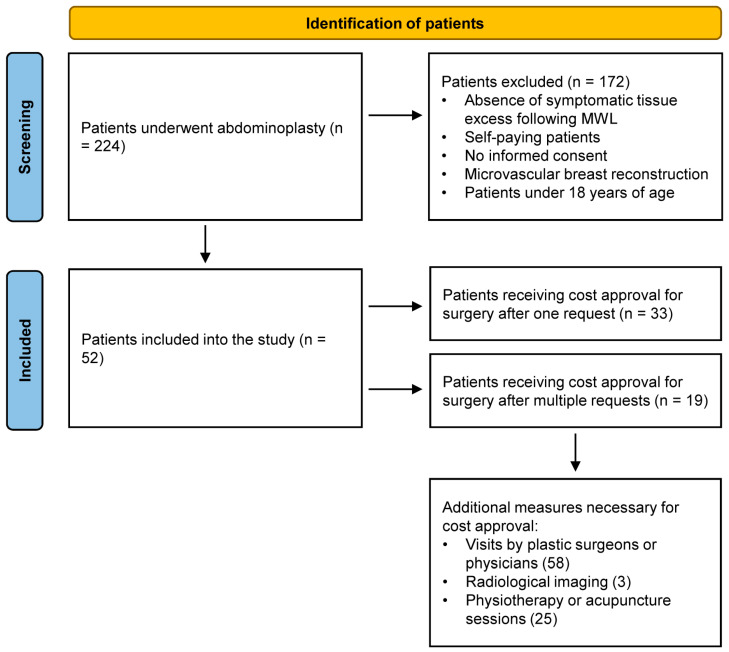
Study flow chart.

A total of 224 patients underwent abdominoplasty during the study period. Of these, 172 patients were excluded for not meeting the inclusion criteria. Consequently, 52 patients were included in the study (Figure 1). The baseline characteristics of the patients are summarized in Table 3, showing no statistically significant differences.

Of the total, 33 patients obtained cost approval after a single request (single-request group), whereas the remaining 19 patients required multiple requests (multiple-request group), i.e., two, three, and four requests for 14, 4, and 1 patient/s, respectively.

Regarding health insurance type, the majority of individuals (*n* = 46) had only the compulsory basic insurance (class III). The remaining patients (*n* = 6) had private or semi-private complementary insurance (class I or II, i.e., the most expensive health insurance). The percentage of cost coverage was categorized as 100% for full coverage and 75% or 50% for partial cost coverage provided by the HIC, which occurred for three patients in the single-request group (Table 4).

**Figure 2:**
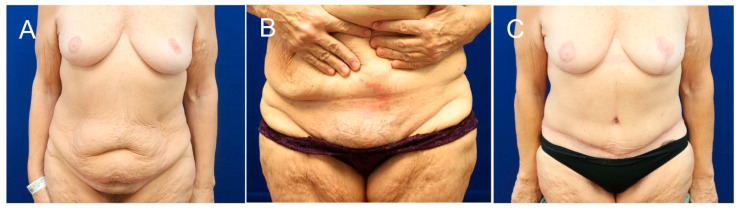
(**A**–**C**) Clinical example of abdominal tissue excess after MWL in a 59-year-old patient, presenting with a large abdominal fat apron. (**B**) Note the skin rash directly at the panniculus fold despite daily local treatments.

Both groups were comparable in terms of clinical signs and symptoms associated with abdominal tissue excess following MWL (Table 5). The most common conditions observed in both groups were skin affections (Figure 2), followed by functional impairments and psychological disorders (Table 5). All patients attempted to manage their skin conditions conservatively with regular or antibiotic-containing creams and, at times, systemic antibiotic therapy, with no real lasting benefits. The Pittsburgh Rating Scale was applied in 48 out of 52 patients with 4 cases missing preoperative photographs. In the single-request group (*n* = 30), 1 patient was classified as grade 1, 14 as grade 2, and 15 as grade 3. In the multiple-request group (*n* = 18), 2 patients were classified as grade 1, 11 as grade 2, and 5 as grade 3. Among the 52 patients, 34 consulted a board-certified plastic surgeon primarily due to skin rashes and other skin affections, 11 mainly for functional impairments, and 7 for psychological issues.

**Figure 3:**
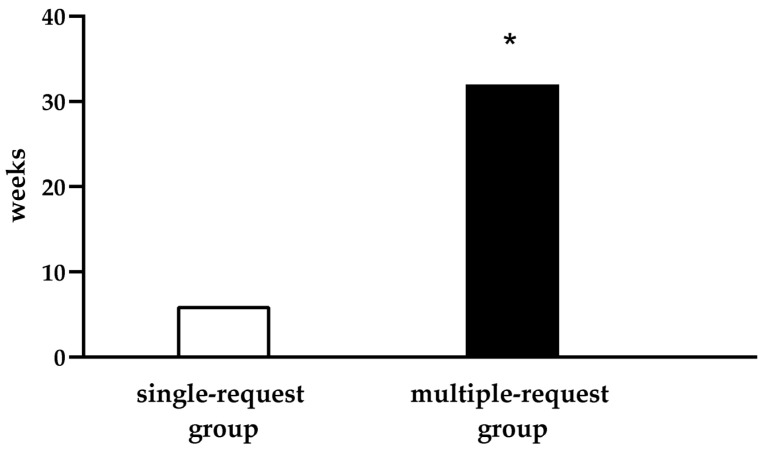
Mean time (in weeks) required for HIC’s cost approval in the single- and multiple-request group. * *p* < 0.05.

Moreover, the mean duration of the approval process, defined as the time from the first reimbursement request to the receipt of final approval, was 15 weeks, with a range of 1 to 103 weeks. Specifically, the mean time to obtain reimbursement confirmation for patients in the multiple-request group was 6.4-fold higher when compared to the single-request group. This reflects a significant additional time of approximately 26 weeks on average for patients requiring multiple requests (*p* < 0.05) (Figure 3).

During the entire period from the first request to cost approval, the multiple-request group required a significantly higher number of out-patient visits, prolonged local skin therapy, physiotherapy and acupuncture sessions when compared to the single-request group (*p* < 0.05) (Table 6). A more detailed analysis of the multiple-request group (*n* = 19) revealed that from the initial rejection of cost approval to the final acceptance, all 19 patients were re-evaluated at least once by their plastic surgeon in order to resubmit the reimbursement request: 14 patients required 1 re-evaluation, 4 patients needed 2 additional visits, and 1 patient underwent 3 additional visits. Furthermore, during this period, 15 of the 19 patients required additional out-patient visits with physicians other than plastic surgeons. Specifically, 7 patients had 1 additional visit, 2 patients had 2 visits, and 6 patients required 3 or more additional examinations. The type of additional consultations is reported in Table 7. In total, 33 additional out-patient visits were performed in the multiple-request group between the initial refusal and the final acceptance. Within this group of patients, 3 abdominal imaging procedures, consisting of 1 CT-scan and 2 ultrasounds, were conducted. Furthermore, 3 patients underwent additional physiotherapy or acupuncture after the rejection, i.e., 22 additional physiotherapy sessions and 3 additional acupuncture sessions. Accordingly, all additional procedures in the multiple-request group generated significant ‘extra’ costs per patient (*p* < 0.05) compared to the single-request group (Table 8).

Regarding surgery, no statistically significant differences were observed between the two groups in terms of type of surgical procedure, weight (kg) and surface area (%) of resected abdominal tissue as shown in detail in Table 9. However, information on the resected tissue weight was unavailable for one individual. The mean postoperative hospital stay was 5 days. Notably, 15 patients underwent 17 self-funded concomitant body contouring procedures during the session of abdominoplasty (Table 9). Among them, 10 (67%) applied for cost coverage, which was denied.

Finally, all 52 patients stated that the procedure fully resolved their clinical and psychological issues, met their expectations regarding the overall outcome, and that they would undergo the same surgery again. Ultimately, surgery resulted in a significant improvement in QoL, with a reduction in discomfort and/or pain from very high levels of 9.6 (range 7–10) before surgery to very low levels of 0.5 (range 0–6) after surgery (*p* < 0.05), with no statistically significant differences between the two groups.

## 4. Discussion

Abdominoplasty is a well-established surgical procedure to address symptomatic abdominal tissue excess following MWL [11]. Surgical treatment has been proven effective in managing permanently skin-related conditions, improving hygiene, addressing psychological disorders and enhancing functional status [22,23,24,25,26]. Although the removal of excess tissue is often considered esthetic, the symptoms reported by post-MWL patients are predominantly functional, representing a condition with a ‘value of disease’ [23]. Therefore, when conservative treatments fail, excision of excess abdominal tissue should be deemed medically necessary and, as such, covered by insurance [23].

Despite the well-documented benefits and medical necessity, a lack of insurance coverage often limits access to body contouring surgeries. Studies indicate that only 5–7% of post-bariatric patients that meet the eligibility criteria, ultimately undergo the surgical intervention [27]. In Switzerland, eligibility for reimbursement is determined by the HIC’s physician based on Swiss DRG criteria (Table 1). Approval criteria for abdominoplasty include documented functional limitations, abdominal scar pain, and the need for a symptomatic rectus diastasis repair. Notably, according to these criteria, skin disorders alone do not constitute a valid indication for surgery if they can be effectively managed conservatively [18]. Furthermore, psychological distress alone does not justify cost coverage for surgery unless exceptional circumstances are present. Additionally, a largely ‘emptied’ abdominal apron is a prerequisite for cost assumption [18]. Although the Swiss DRG criteria are relatively well-defined, discrepancies persist between board-certified plastic surgeon’s indications and the HIC’s reimbursement decisions, as demonstrated in this study in 37% of cases.

Similar inconsistencies occur in other countries, where reimbursement criteria are often misinterpreted or vary across insurers, complicating the approval process [28,29,30]. For instance, in the United States of America, Gallagher observed that insurance carriers hesitate to approve panniculectomy to resect abdominal tissue excess or an apron due to misunderstandings about patient’s functional limitations. In such cases, comprehensive documentation by healthcare professionals, including wound, ostomy, and continence nurses, pain specialists, and therapists, is pivotal to support reimbursement claims [31]. In 2008, Gurunluoglu highlighted that although third-party payer’s criteria align with the American Society of Plastic Surgeons standards, they are harder to meet [32]. Similarly, Dreifuss and Rubin identified a discrepancy between plastic surgeon’s assessments of abdominoplasty eligibility and insurer’s requirements, recommending increased HIC’s physician involvement in refining insurance coverage guidelines for body contouring procedures after MWL [33]. In Europe, a survey across 20 out of 27 EU National Health Systems found that access criteria for post-bariatric body-contouring surgeries vary substantially, mostly including a stable weight and a BMI maintained inferior to specific levels [34]. More recently, the Swiss DRG criteria for the treatment of symptomatic breast hypertrophy have been criticized for their susceptibility to subjective interpretation [19].

The results of the present study are in line with these observations. Indeed, all 19 patients whose cost coverage was initially refused ultimately received approval after resubmission/s. This suggests that their original application already met the established criteria. Moreover, the majority of the included patients presented moderate to severe abdominal deformity according to the Pittsburgh Rating Scale, supporting the appropriateness of surgical indication in both single- and multiple-request group [21]. Inconsistencies may arise from the increasing prevalence of borderline cases, combined with the fact that HIC physicians are rarely specialists in the field or examine patients directly, instead relying solely on the information and documentation submitted with reimbursement requests, which always leaves some room for interpretation. This highlights the importance of comprehensive and accurate documentation in facilitating approval. Consistently, a previous study showed that including certain objective criteria (e.g., medical indications, a high BMI before bariatric surgery, high BMI reduction, and mechanical restrictions) in the request letters, or submitting additional application letters to the HICs, was associated with a positive response, yet decisions remained largely unpredictable [35].

To evaluate the impact of reimbursement denials, two groups were compared: patients who received immediate approval and those who required multiple submissions. Both groups were comparable in terms of baseline and perioperative characteristics. Moreover, there were no significant differences in signs and symptoms following MWL, underscoring the accuracy of the board-certified plastic surgeon’s initial indication for surgery. The multiple denials resulted in a significant loss of time and resources, with additional costs of approximately CHF 715 per patient, corresponding to 6.2% of the total cost of the surgery. In both groups, surgery effectively resolved MWL-related conditions. Patient well-being improved significantly, with a mean reduction in discomfort and/or pain from very high levels of 9.6 (range 7–10) pre-surgery to 0.5 (range 0–6) post-surgery, emphasizing the significant impact of abdominoplasty.

Although this study provides the first evidence of the clinical and economic implications of reimbursement delays for patients with symptomatic abdominal tissue excess after MWL in Switzerland, several limitations should be mentioned. Firstly, the retrospective nature may have introduced information bias due to the reliance on pre-existing records, which could be incomplete or inconsistent. Moreover, the relatively small sample size (*n* = 52) may limit the statistical power and generalizability of the results. However, a post hoc power analysis revealed that the observed difference in time (in weeks) required for cost approval between the two groups had a high statistical power of almost 99%. Another limitation of the present study is the use of a non-validated, study-specific questionnaire. However, this choice was dictated by the retrospective nature and the primary objective of documenting the pathological condition of patients after MWL rather than quantifying their postoperative improvements. A formal cost-effectiveness analysis at the healthcare system level was beyond the scope of this study and should be addressed in future research. Finally, selection bias may arise from the fact that baseline characteristics of excluded patients were not assessable, also some eligible patients with abdominal tissue excess, initially seen in our department, ultimately decided to undergo surgery at their own expenses elsewhere rather than wait for the eventual HIC’s acceptance.

Accordingly, a recent systematic review on cosmetic tourism reported Switzerland as the second most frequent country of origin from which patients sought cosmetic surgery abroad, with abdominoplasty being the second most common procedure [36]. Although not directly assessed in our study, restrictive reimbursement policies, less expensive prices, and knowledge of potential cost coverage in the event of complications in the home country could be factors among others explaining why some Swiss patients seek surgery abroad. Taken together, abdominoplasty significantly improved QoL in all included patients, with no differences between the groups. However, initial denial of cost coverage despite clear surgical indications, led to treatment delays, prolonged symptomatology, and increased healthcare costs. Therefore, strengthening the collaboration between HICs and plastic surgeons to better define clear recommendations, could help promote more consistent, case-by-case, evaluations already at the initial stages of the reimbursement request, potentially shortening the time required for cost approval. A step towards this goal can be the standardization of a ‘minimum’ set of documentation with well-defined characteristics needed by the HIC to adequately evaluate the case in absence of the patient. Nevertheless, given the abovementioned limitations, these findings should be corroborated by further studies involving larger cohorts. It is also important to note that these results specifically refer to healthcare systems that provide reimbursement for abdominoplasty performed for symptomatic abdominal tissue excess.

## Figures and Tables

**Table 1:** Criteria to be met for health insurance companies (HICs) in Switzerland to reimburse the costs for abdominoplasty in patients suffering from symptomatic abdominal tissue excess after massive weight loss (MWL).

Criteria
abdominal tissue excess that causes functional limitations or scar pain
association with symptomatic rectus diastasis, possibly confirmed by instrumental examination
the abdominal apron must be largely ‘emptied’ following weight loss
conservative measures (e.g., drugs, physiotherapy or local treatments) have remained ineffective

**Table 2:** TARMED-based prices for additional diagnostic and therapeutic measures.

Service	TARMED-Based Prices (CHF)
- evaluation by board-certified plastic surgeon	136.20
- evaluation by other board-certified specialist	240.30
- abdominal CT scan	239.00
- abdominal ultrasound	146.00
- single session of physiotherapy	69.30
- single session of acupuncture	90.20

CT = computed tomography.

**Table 3:** Baseline patient’s characteristics in the single- and multiple-request group. Number of patients (*n*); percentage; mean ± standard deviation (SD). No significant differences between the groups.

Baseline Patient’s Characteristics	Single-Request Group (*n* = 33)	Multiple-Request Group (*n* = 19)	Total
gender			
female (*n*, %)	23 (69.7)	15 (78.9)	38 (73.1)
male (*n*, %)	10 (30.3)	4 (21.1)	14 (26.9)
age at surgery (years ± SD)			
comorbidities (% ± SD)	50.9 ± 11.3	46.0 ± 11.6	49.1 ± 11.6
smoking (*n*, %)	5 (15.1)	1 (5.2)	6 (11.5)
arterial hypertension (*n*, %)	4 (12.1)	0 (0.0)	4 (7.7)
diabetes (*n*, %)	3 (9.0)	1 (5.2)	4 (7.7)
dyslipidemia (*n*, %)	1 (3.0)	0 (0.0)	1 (1.9)
chronic kidney disease (*n*, %)	0 (0.0)	0 (0.0)	0 (0.0)
sleep apnea syndrome (*n*, %)	4 (12.1)	1 (5.2)	5 (9.6)
height (cm ± SD)	166.8 ± 9.5	166.5 ± 9.3	166.7 ± 9.3
BMI before MWL (kg/m^2^ ± SD)	46.1 ± 8.4	47.3 ± 5.0	46.6 ± 7.3
BMI at the time of abdominoplasty (kg/m^2^ ± SD)	27.8 ± 4.9	29.5 ± 4.2	28.4 ± 4.7
weight before MWL (kg ± SD)	131.9 ± 30.4	132.9 ± 14.9	132.2 ± 25.6
weight at the time of abdominoplasty (kg ± SD)	75.9 ± 13.9	79.2 ± 10.0	77.1 ± 12.6
ideal weight (kg ± SD)	61.4 ± 7.0	61.2 ± 6.8	61.4 ± 6.9
excess of weight before MWL (kg ± SD)	70.5 ± 25.6	71.8 ± 12.6	70.9 ± 21.7
percentage of weight loss (% ± SD)	79.6 ± 16.9	75.1 ± 11.5	78.0 ± 15.2
methods for achieving MWL (*n*, %)			
gastric bypass	23 (69.7) ^+^	12 (63.1)	35 (67.3) ^+^
sleeve gastrectomy	9 (27.3) ^+^	3 (15.8)	12 (23.0) ^+^
gastric banding	0 (0.0)	1 (5.3)	1 (1.9)
diet	3 (9.0)	3 (15.8)	6 (11.5)
duration of weight stability after MWL (months ± SD)	12.7 ± 10.6	9.3 ± 5.6	11.2 ± 8.8

^+^ Two patients underwent both gastric bypass and sleeve gastrectomy. BMI = body mass index.

**Table 4:** Health insurance characteristics. Number of patients (percentage). No significant differences between the groups. However, within each group there was a statistically significant difference between the number of patients with compulsory (class III) and complementary (class II or I) health insurances. * *p* < 0.05.

Health Insurance	Single-Request Group (*n* = 33)	Multiple-Request Group (*n* = 19)	Total
class I	1 (3.0)	2 (10.5)	3 (5.7)
class II	3 (9.0)	0 (0.0)	3 (5.7)
class III	29 (87.9) *	17 (89.5) *	46 (88.5)
class I–III	33 (100.0)	19 (100.0)	52 (100.0)
**Cost Coverage**			
100%	30 (90.9)	19 (100.0)	49 (94.2)
75%	1 (3.0)	0 (0.0)	1 (1.9)
50%	2 (6.1)	0 (0.0)	2 (3.8)
50–100%	33 (100.0)	19 (100.0)	52 (100.0)

**Table 5:** Signs and symptoms associated with abdominal tissue excess following MWL, in the single- and multiple-request group. Number of patients (percentage). No significant differences between the groups.

Signs and Symptoms	Single-Request Group (*n* = 33)	Multiple-Request Group (*n* = 19)
skin affections	31 (93.9)	19 (100.0)
functional impairments	14 (42.4)	5 (26.3)
psychological disorders	12 (36.4)	7 (36.8)
symptomatic rectus diastasis	7 (21.2)	6 (31.6)
severe rectus diastasis ^+^	1 (3.0)	1 (5.3)
abdominal hernias	8 (24.2)	5 (26.3)
back pain	3 (9.1)	5 (26.3)

^+^ Rectus diastasis is internationally classified as mild (< 3 cm), moderate (3–5 cm, surgery is possible), and severe (> 5 cm, surgery is recommended).

**Table 6:** Mean additional diagnostic and therapeutic measures needed per patient between the first request and the final acceptance in the single- and multiple-request group. Mean ± SD. * *p* < 0.05 and ** *p* < 0.001.

Diagnostic and Therapeutic Measures	Single-Request Group (*n* = 33)	Multiple-Request Group (*n* = 19)
visit by plastic surgeons	0.0 ± 0.0	1.3 ± 0.6 **
visit by specialists other than plastic surgeons radiological imaging	0.0 ± 0.0	1.7 ± 1.6 **
	0.0 ± 0.2	0.2 ± 0.6
physiotherapy and acupuncture sessions	0.0 ± 0.0	1.3 ± 4.2 *
total	0.0 ± 0.2	4.6 ± 4.3 **

**Table 7:** Type of specialist other than plastic surgeon and number of additional visits (N) in the multiple-request group between the initial refusal and the final acceptance.

Specialist	N in the Multiple-Request Group
general practitioner	9
endocrinologist	6
psychiatrist	6
dermatologist	5
neurologist	2
psychologist	2
gastroenterologist	1
general surgeon	1
orthopedic surgeon	1
total	33

**Table 8:** Mean ‘extra’ costs per patient in CHF for diagnostic and therapeutic measures that occurred between the first request and the final acceptance in the single- and multiple-request group. Cost difference between the two groups. Mean ± SD; * *p* < 0.05 and ** *p* < 0.001.

Diagnostic and Therapeutic Measures	Single-Request Group (*n* = 33)	Multiple-Request Group (*n* = 19)	Cost Difference
visits by plastic surgeons ^+^	0.0 ± 0.0	179.2 ± 79.3 **	179.2 ± 18.2
visits by other specialists	0.0 ± 0.0	417.4 ± 374.4 **	417.4 ± 85.9
radiological imaging	4.4 ± 25.4	27.9 ± 68.6	23.5 ± 16.4
physiotherapy and acupuncture	0.0 ± 0.0	94.5 ± 292.1 *	94.5 ± 67.0
total	4.4 ± 25.4	718.9 ± 446.3 **	714.5 ± 102.5

^+^ The cost of the first visit is excluded.

**Table 9:** Perioperative patient’s characteristics in the single- and multiple-request group. Number of patients (percentage); mean ± SD.

Perioperative Patient’s Characteristics	Single-Request Group (*n* = 33)	Multiple-Request Group (*n* = 19)	Total
type of ‘abdominoplasty’			
abdominoplasty	25 (75.7)	6 (31.6)	31 (59.6)
belt lipectomy	7 (21.2)	12 (63.1)	19 (36.5)
panniculectomy	1 (3.0)	1 (5.2)	2 (3.8)
weight of resected abdominal tissue (kg) ^+^	1.8 ± 1.1	2.2 ± 0.9	1.9 ± 1.0
percentage of resected surface (%) ^+^	4.5 ± 0.0	5.6 ± 0.0	4.9 ± 0.0
self-funded concomitant surgeries	11 ± 0.7	6 ± 0.5	17 ± 0.6
length of hospital stays (days)	4.9 ± 1.9	5.2 ± 1.7	5.1 ± 1.8

^+^ These values were calculated for 18 patients in the multiple-request group, as the weight of the resected abdominal tissue was unavailable for one patient.

## Data Availability

The data that support the findings of this study are available from the corresponding author upon reasonable request.

## Abbreviations

The following abbreviations are used in this manuscript:

BMI	Body Mass Index
CT	Computed Tomography
HIC	Health Insurance Company
MWL	Massive Weight Loss
NRS	Numerical Rating Scale
QoL	Quality of Life
Swiss DRG	Swiss Diagnosis Related Group
